# Assessment of work-related asthma prevalence, control and severity: protocol of a field study

**DOI:** 10.1186/s12889-016-3824-0

**Published:** 2016-11-16

**Authors:** Hermine Mével, Valérie Demange, Emmanuelle Penven, Christian Trontin, Pascal Wild, Christophe Paris

**Affiliations:** 1 INRS, Institut National de Recherche et de Sécurité, Vandoeuvre-lès-Nancy, France; 2 EA7298 INGRES, Université de Lorraine, Nancy, France; 3 Occupational Diseases Department, University Hospital, Vandoeuvre-lès-Nancy, France

**Keywords:** Asthma control, Asthma diagnosis, Asthma quality of life, Asthma severity, Occupational epidemiology, Occupational asthma, Work-related asthma, Work-exacerbated asthma, Medico-economical evaluation

## Abstract

**Background:**

There are still uncertainties regarding the respective prevalence, diagnosis and management of occupational asthma (OA) and work-exacerbated asthma (WEA). There is as yet no standardized methodology to differentiate their diagnosis. A proper management of both OA and WEA requires tools for a good phenotyping in terms of control, severity and quality of life in order to propose case-specific therapeutical and preventive measures. Moreover, there is a lack of knowledge concerning their actual costs.

**Methods:**

This project aims at comparing 3 groups of asthmatic subjects at work: subjects with OA, with WEA, and with non-work-related asthma (NWRA) in terms of control, severity and quality of life on the one hand, and estimating the prevalence of OA, WEA and NWRA in active workers and the economic costs of OA and WEA, on the other hand. Control will be assessed using the Asthma Control Test questionnaire and the daily Peak Exploratory Flow variability, severity from the treatment level, and quality of life using the Asthma Quality of Life Questionnaire. A first step will be to apply a standardized diagnosis procedure of WEA and OA. This study includes an epidemiological part in occupational health services by volunteering occupational physicians, and a clinical case-study based on potentially asthmatic subjects referred to ten participating University Hospital Occupational Diseases Departments (UHODD) because of a suspected WRA. The subjects’ characterization with respect to OA and WEA is organized in three steps. In Step 1 (epidemiological part), occupational physicians screen for potentially actively asthmatics through a questionnaire given to workers seen in mandatory medical visit. In step 2 (both parts), the subjects with a suspicion of work-related respiratory symptoms answer a detailed questionnaire and perform a two-week OASYS protocol enabling us, using a specifically developed algorithm, to classify them into probably NWRA, suspected OA, suspected WEA. The two latter groups are referred to UHODD for a final harmonized diagnosis (step 3). Finally, direct and indirect disease-related costs during the year preceding the diagnosis will be explored among WRA cases, as well as these costs and the intangible costs, during the year following the diagnosis.

**Discussion:**

This project is an attempt to obtain a global picture of occupational asthma in France thanks to a multidisciplinary approach.

**Electronic supplementary material:**

The online version of this article (doi:10.1186/s12889-016-3824-0) contains supplementary material, which is available to authorized users.

## Background

### Rationale

While occupational asthma (OA) has been identified a long time ago, the concept work-exacerbated asthma (WEA) has been defined in the literature more recently [1–3]. The American College of Chest Physicians (ACCP) committee defines work-related asthma (WRA) as “the broad term that refers to asthma that is exacerbated or induced by inhalation exposures in the workplace” [1]. WRA includes both occupational asthma (OA) and work-exacerbated asthma (WEA). The ACCP committee defines OA as “asthma caused by exposure to sensitizing agents and/or irritants in the workplace” [1]. WEA is defined by the American Thoracic Society (ATS) as “pre-existing or concurrent asthma that is worsened by workplace conditions” [2]. An ATS statement [3] estimates that 15% of adult asthma can be attributed to occupation (OA). With respect to WEA, knowledge is comparatively less precise, leading to uncertainty regarding the prevalence (a rough estimate of 21.5% of adults with asthma has however been given [2]), diagnosis and management. In 2014, Aasen et al. [4] proposed a general WRA diagnostic approach based on the patient interview and repeated PEF (peak expiratory flow) or FEV (forced expiratory volume) measurements, at work and away from work. The final etiological diagnosis for OA relies on immunological testing with the specific inhalation challenge (SIC), considered as the gold standard. When SIC is not possible, comparative non-specific bronchial challenges and bronchial inflammation measurements at work and out of work are usually considered. However, despite some available guidelines [5–9] there is as yet no standardized methodology in the different diagnoses within WRA. The more precise definition of asthma phenotypes [10] based on stricter severity and control criteria proposed recently has led to changes in the clinical management of asthma. Asthma control has two domains: asthma symptom control, assessed from the frequency of daytime and nighttime asthma symptoms and short-term relief treatment use, as well as from activity limitation on the one hand, and future risk of adverse outcomes, assessed by lung function, on the other hand. The level of asthma control is determined by the subject’s genetic background, underlying disease processes, the treatment taken, and environmental and psychosocial factors. Asthma severity is classified from “mild” to “severe”, and assessed by the level of treatment required to control symptoms and exacerbations [10–13]. Health related quality of life in asthmatics, based on the patient’s perception, is a subjective aspect of asthma control and severity and must be considered to have a complete picture of the burden of asthma [14–17]. Thus a better phenotyping of WRA in terms of control and severity, but also quality of life, is critical if we want to propose case-specific therapeutical and preventive measures. Finally, although WRA is recognized as a heavy burden in the developed countries, there is a lack of knowledge concerning its actual costs. Considering only costs related to compensation considerably underestimates social and occupational damages of WRA. Two studies [18, 19] focusing on the cost of OA have been conducted and found the social and financial costs of OA to be very high. One study [20] found that asthma-related costs of both WEA and OA were 10 fold greater than the costs related to NWRA.

### Study objectives

**Fig. 1:**
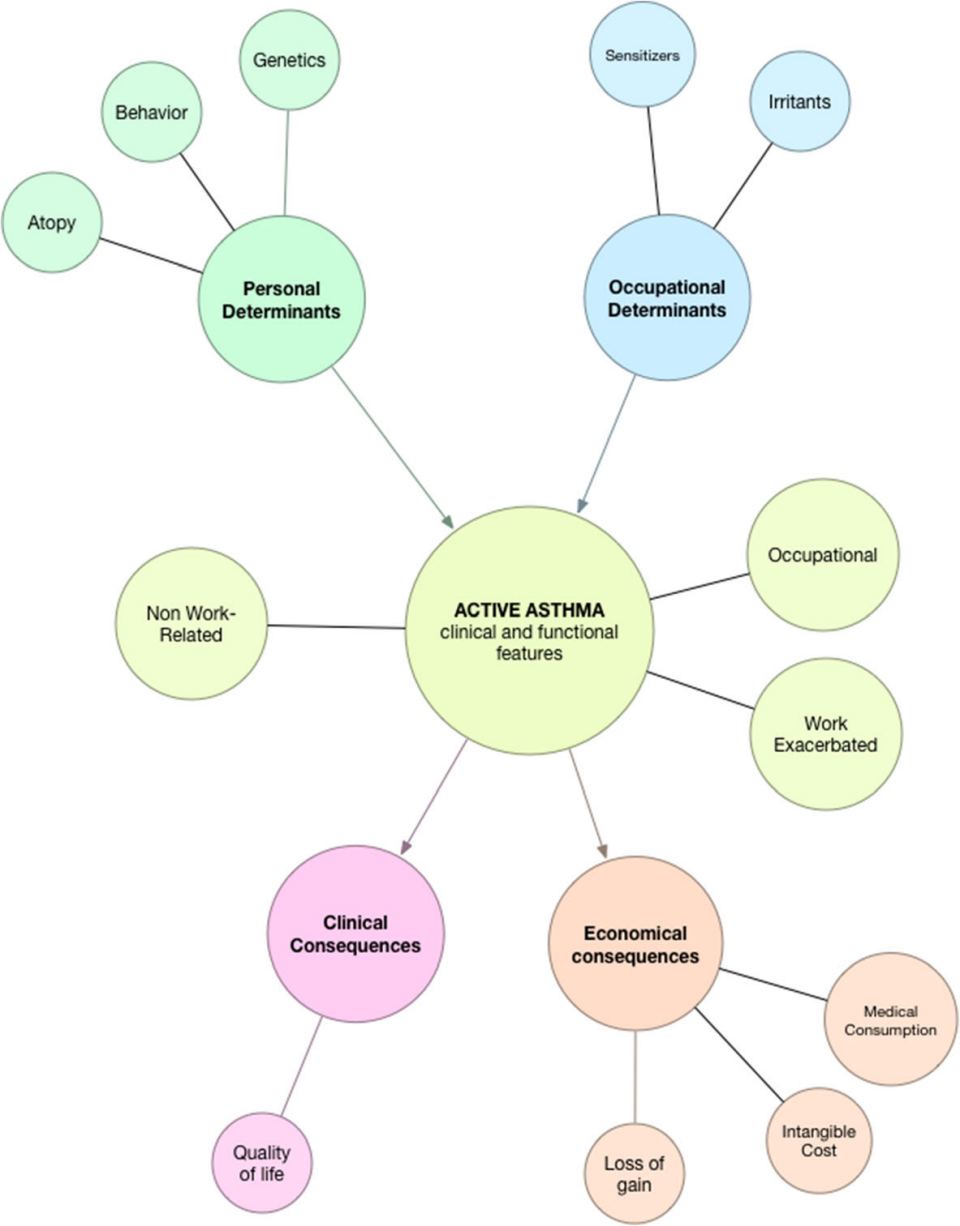
Project concepts

The project presented in this paper (the Arpeige project) encompasses several concepts to describe the disease of asthma in active workers (Fig. 1). The goals of this study are:

- To define and characterize asthma phenotypes in terms of control, severity and quality of life, between 3 groups of working asthmatic subjects: (a) subjects with OA, (b) subjects with WEA, and (c) subjects with non-work-related asthma (NWRA);
- To estimate the prevalence of OA, WEA and NWRA in the workers and to compare their occupational exposures;
- To devise and apply a standardized diagnosis procedure of WEA and OA with or without SIC;
- To estimate the economic costs of OA and WEA, encompassing direct costs (healthcare and non-healthcare) as well as indirect costs (productivity losses and a contingent evaluation: “willingness to pay”).

## Methods

In France, employers are legally requested to monitor on a regular basis their employees’ work-related health. This follow up is done by occupational physicians usually organized in occupational health services, funded by employers, and concerns all employees whether or not any presumed occupational risk factors might exist. These occupation physicians usually cover several industrial sectors. In addition to this surveillance system, University Hospital Occupational Diseases Departments (UHODD) assess the occupational origin of health issues of patients referred by occupational physicians, general practitioners or other health specialists.

The present study is based on these two structures.

### Study design

This study is comprised of an epidemiological part and a clinical part. The epidemiological part includes any volunteer subject aged from 16 to 65 active workers, seen in mandatory medical examination over a 2 week period by occupational physicians who agreed to participate. The clinical part includes any suspected WRA cases referred to the 10 participating UHODD as well as the asthmatic subjects identified in the epidemiological part of the study.

### Sample size

We expect each UHODD to recruit between 20 and 30 subjects per year within two years, totaling 400 subjects with work-related asthma. We expect to recruit 100 occupational physicians, each following an average number of 100 workers in two weeks. Assuming an asthma prevalence of 5% in the working population, the number of identified asthmatics within 10,000 screened subjects is thus expected to be 500, among which 75 work-related asthmatics, applying the percentages mentioned in the introduction: 15% (OA) + 21.5% (WEA). This allows a precision of 4.6–5.4% in the estimation of the prevalence of working asthmatics and a global precision of 32.2–41.0% in the percentage of WRA. Assuming a clinical feature (e.g., intermittence of symptoms) with a 75% prevalence in one group of asthmatics (of NWRA, OA and WEA), these numbers allow to detect a significant difference of −8% to +7% with an 80% power.

### Data collection strategy for the identification of WRA

**Fig. 2:**
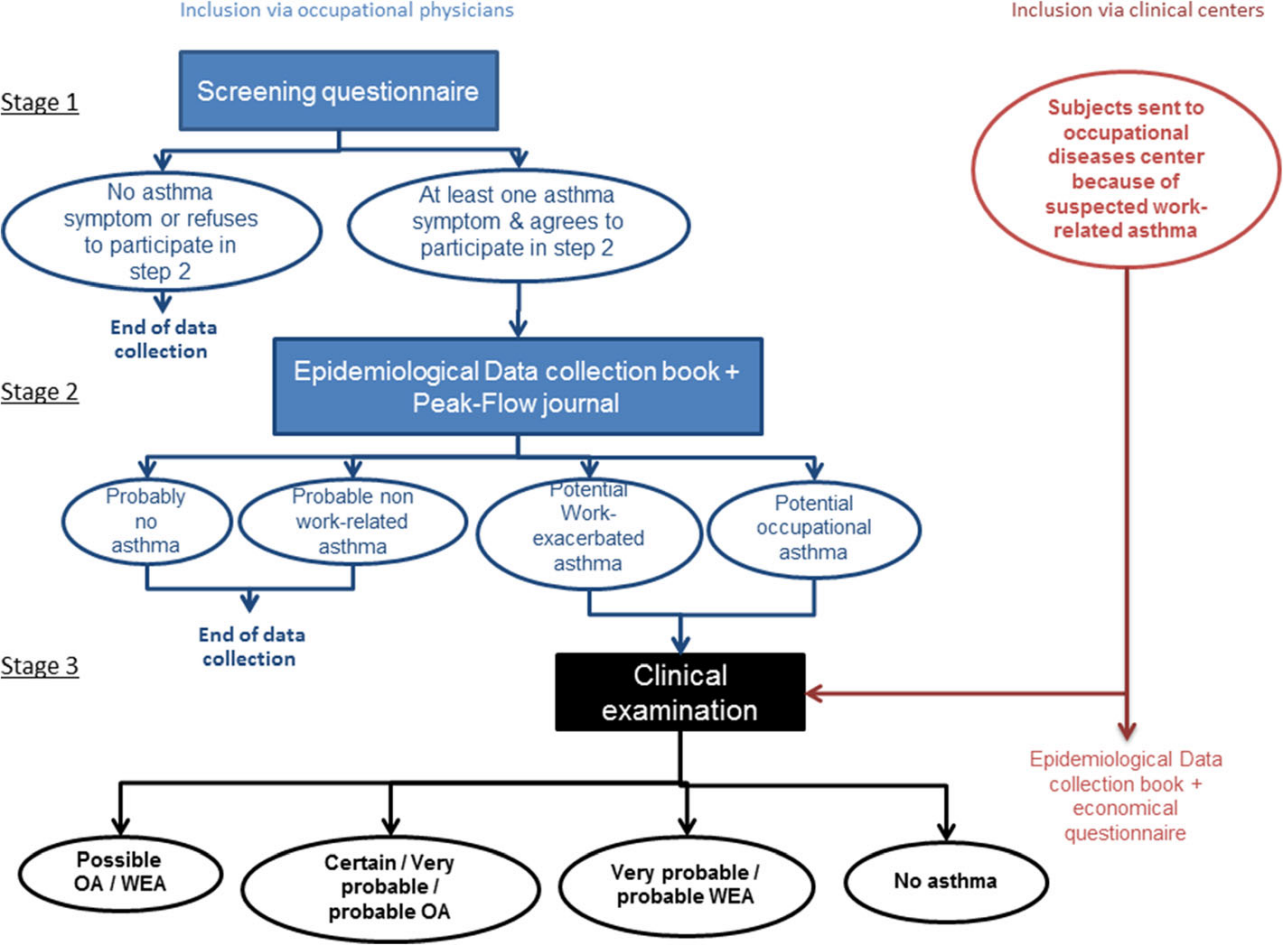
Study design. Abbreviations: OA occupational asthma, WEA work-exacerbated asthma, NWRA non work-related asthma

The characterization of the study subjects with respect to WRA is organized in 3 steps (Fig. 2). The data collected for each concept of the project are summarized in Table 1.

**Table 1:** Concepts and collected data

Concepts		Collected data
PERSONAL DETERMINANTS	genetics	
	behavior	tobacco status
	atopy	skin prick tests or serological specific IgE to common allergens
OCCUPATIONAL DETERMINANTS	occupational exposures	occupational history occupational exposures to sensitizers and irritants (task based questionnaire)
	occupational sensitization	SPT, specific IgE, Specific Inhalation Challenge to occupational allergens
ACTIVE ASTHMA: WEA, OA, NWRA	control	clinical control: score of the Asthma Control Test questionnaire
		respiratory functional control: PEF daily variation
	severity if well-controlled asthma	GINA’s steps of treatment
CLINICAL CONSEQUENCES	quality of life	Asthma Quality of Life questionnaire score
ECONOMICAL CONSEQUENCES	consumption due to asthma	medical care consumption and improvements in the housing environment
	loss of gain due to asthma	job changes, workshift changes, sick leaves
	direct intangible costs	phone interview – scenario

Step 1 (*a priori* classification) is a screening step carried out among active workers followed for the mandatory health examinations. It is based on a quick self-administered screening questionnaire (one recto-verso page), adapted from the European Community Respiratory Health Survey [21] asthma questionnaire. It comprises items regarding asthma antecedents (childhood asthma), active asthma symptoms, and smoking status. Additional questions about asthma symptoms occurring at work or hours following work shift are adapted from the Occupational Asthma Screening Questionnaire–11 items (OASQ-11) previously evaluated in a clinical setting by Pralong et al. [22]. The collected demographic data items are sex, age and occupation. A subject is considered as potentially asthmatic, and invited to undertake the next step if s/he answers positively to at least one of the questions on asthma symptoms (see Additional file 1). The subject’s symptoms are considered as potentially work-related if at least one of them occurs or is exacerbated at work (or a few hours after work) and/or if they improve or disappear when away from work. Each worker is then classified as “probably not actively asthmatic”, “potentially actively asthmatic” or “potentially actively asthmatic with work-related symptoms”, based on the answers to the screening questionnaire.

Step 2 (intermediate classification): when a subject with a potential active asthma (work-related or not) identified in step 1, gives his consent, the occupational physician invites him to take home a peak flow meter (Oasys II protocol) and a data collection book, consisting of:

- a medical questionnaire adapted from standardized questionnaires [23–25], with a detailed asthma and allergy personal and familial history, asthma treatment, link between symptoms and work,
- a detailed job history and questions about a possible change in job status due to an asthma or respiratory condition,
- a task-based questionnaire, based on the tasks exposing to occupational allergens and irritants that are likely to induce or aggravate asthma, listed in the French national occupational disease surveillance and prevention network RNV3P and in the literature. The starting date and the frequency of each task are asked,
- the Asthma Control Test (ACT) [26] to assess the asthma control level,
- the Asthma Quality of Life Questionnaire (AQLQ) [14] to assess asthmatic subjects’ quality of life,
- a list of every drug prescribed for their asthma along with the dose and frequency of intake.

The same procedure is applied to any subject with suspected WRA referred to the UHODD participating in the study. The Oasys protocol is adapted as follows: 4 PEF measurements a day over a 2-week period in case of epidemiological recruitment and over a 4 to 6-week period in case of clinical recruitment. The OASYS-2 tool [27, 28], a validated diagnostic aid for WRA, is used to generate PEF summary plots and indexes. The used indexes and their interpretation are summarized in Table 2.

**Table 2:** Oasys protocol and interpretation

Part of the study	Duration of PEF measurements period	Used indexes	Interpretation
Epidemiological	2 weeks	20% daily variation of PEF	Active asthma
Clinical	4–6 weeks	Oasys score	>2.5 : in favor of OA 1.5–2.5 in favor of WEA <2.4: in disfavor of WEA

Based on all data collected at the 2 previous steps, the included potential asthmatics are then classified by consensus of two occupational physicians from one UHODD and two epidemiologists into 4 categories: “probably no active asthma”, “probably NWRA”, “suspected WEA” and “suspected OA”. Subjects with suspected WRA are invited to participate in step 3 (clinical examinations) in one of the UHODD. Non-work-related asthmatics are those with childhood asthma, without any exposure to known asthmogen and no work-related pattern in symptoms and in the recorded PEFs. If the symptoms appear to be poorly controlled, they are advised to see a physician.

**Fig. 3:**
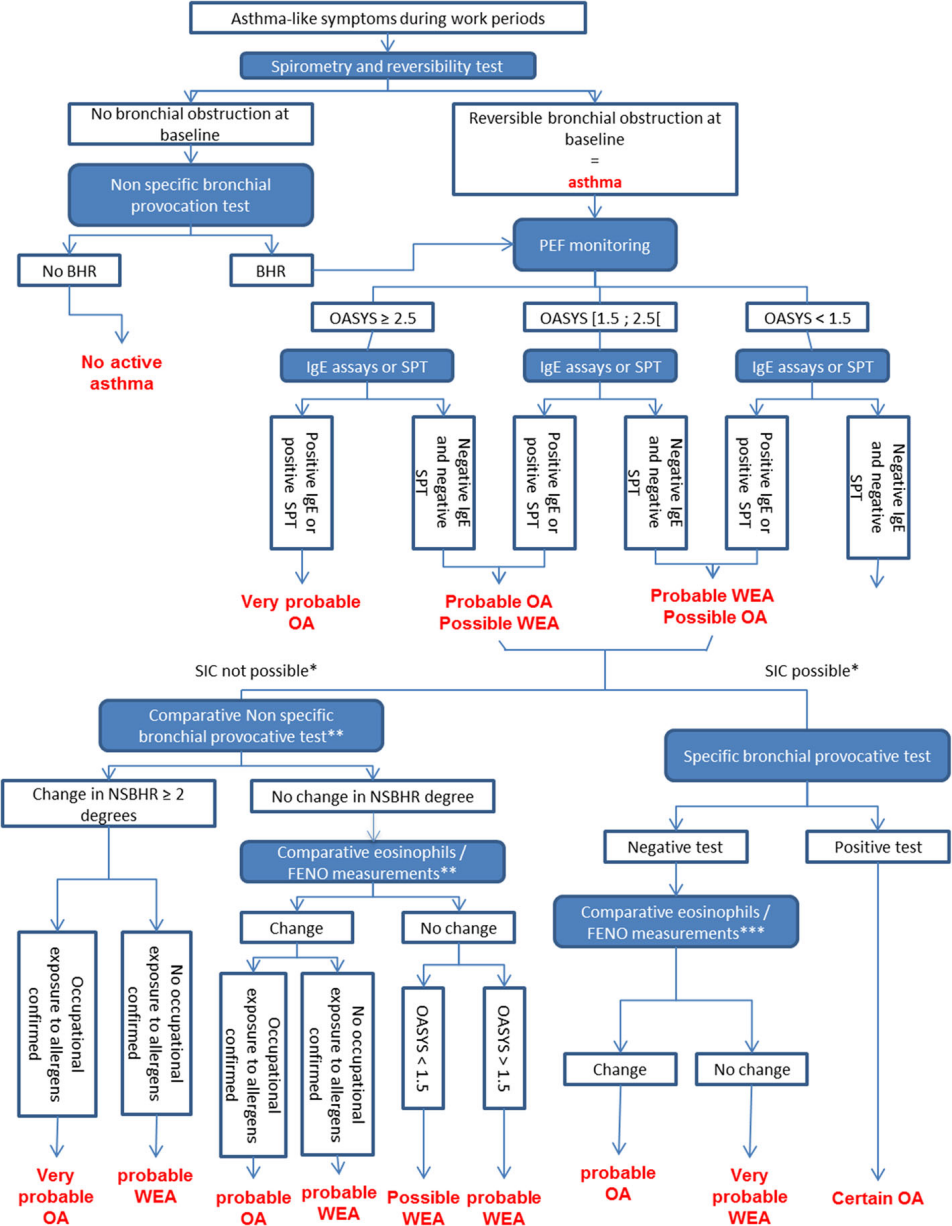
Decision tree for the diagnosis of work-related asthma. BHR: Bronchial hyperresponsiveness; PEF: Peak Expiratory Flow; SPT: Skin Prick-Tests; OA: Occupational asthma; WEA: Work-exacerbated asthma; NWRA: Non Work1Related Asthma; NSBHR: Non Specific Bronchial Hyperresponsiveness; *Specific Inhalation Challenge [33] is done according to the center’s means and if an occupational allergen is identified; **One test after at least 2 consecutive working weeks and another test after at least 10 days away from work; ***between before and after the specific bronchial provocative test

Step 3 (final classification): all suspected WRA patients selected in step 2 will be diagnosed as WEA and OA based on clinical examinations in the UHODD participating. In order to obtain a standardized and reproducible classification, we developed a decisional algorithm summarized in Fig. 3 in two stages based on the latest guidelines on WRA diagnosis approach [5–9, 29]. This algorithm was aimed at being adaptable to the existing diagnostic tools available in the different clinical centers, particularly the possibility to perform SIC or not (Table 3):

**Table 3:** Clinical examination in step 3, according to the diagnosis means

	in all centers	where possible
STAGE 1	• detailed medical interview (occupational exposures, medical history and symptoms) • extended OASYS protocol (4–6 weeks) • skin-prick-tests to common airborne allergens and to potentially allergenic occupational agents • flow-volume curves with beta2 agonist reversibility test • methacholine challenge test • total serum IgE measurements and, • if possible, allergen specific IgE	• exhaled Nitric Oxyde measurements • induced sputum to make a cytological analysis (polynuclear cells, neutrophils and eosinophils)
STAGE 2 (if suspected WRA)	• one or several specific inhalation challenge (SIC) with the potential occupational allergen • one bronchial exposure test at the work station • comparative methacholine challenge tests, in working period and resting period.	• comparative exhaled Nitric Oxyde measurements before and after exposure • induced sputum cytologic analysis before and after exposure

- In a first clinical stage, subjects will be classified into « non-asthmatic », « probably not work-related asthma», « probable WRA » (« probable OA », « probable WEA », « possible OA or WEA ») based on spirometry, reversibility tests, non-specific bronchial challenge test, specific IgE and/or skin prick tests and the OASYS score.
- The possible or probable WRA cases will undergo the second stage, including complementary tests leading to the final “objective” classification as « non-asthmatic », «NWRA », « certain OA », « probable OA » or « probable WEA ».

Finally, this classification will be confirmed or invalidated based on a consensus among specialists of work-related asthma, considering all available data.

### Asthma control assessment

Parallel to the above classification, asthma symptom control is assessed using 3 criteria:

- The ACT (Asthma Control Test) during step 2. It is a simple self-administered, five-point questionnaire. It includes four symptom/reliever questions plus a patient self-assessed level of control. Each item is scored from one (poor control) to five (good control) and the scores added to give a final score that ranges from 5 to 25. Scores of 20–25 are classified as well-controlled asthma, 16–20 as not well-controlled, and 5–15 as very poorly controlled asthma.
- The daily PEF variability: the symptoms are poorly controlled if the daily variability is over 20% at least once per week.

The frequency of severe exacerbations: during Step 3 (clinical examination), the subject is asked how many times they had to see their physician or go to an emergency department because of an exacerbation of their asthma in the past year.

Asthma severity is assessed from the treatment level for well-controlled asthma only [30]. Subjects are classified in four severity levels:

- Intermittent asthma for Step 1,
- Mild persistent asthma for Step 2 treatment,
- Moderate persistent asthma for a Step 3 treatment,
- Severe persistent asthma for a Step 4 or Step 5 treatment.

### Medico-economic evaluation

Disease costs are usually classified in direct and indirect, and are sustained by the subject with WRA and their family, by their employer, and by the Society. Direct costs can be easily converted into economic terms, and are classified in medical costs (diagnosis, drug therapy, medical care, in-patient treatment, etc.) and non-medical costs (patient’s out-of-pocket expenses, like transport, cost of making changes to house…). Indirect costs measure the value of resources lost due to WRA (time off work due to sick leave, reduced productivity at work …). The societal cost of OA, in a cost-of-illness approach, includes both direct and indirect costs. Intangible costs are the value placed on reduced quality of life, pain, suffering… One method of estimating the monetary value of intangible costs is contingent valuation method, a stated preference method based on the elicitation of levels of willingness to pay (WTP). Despite the conceptual debates (valuing pain in monetary terms as there is no real market existing) and the inherent difficulties measuring the WTP (subjective responses, double counting), the estimation of intangible costs is necessary in the individual perspective. In practice, as soon as a diagnosis of WRA is established, subjects at the UHODDs are given a questionnaire comprised of questions regarding, during the past year, the number of physician visits and hospitalizations because of the respiratory symptoms and how much they paid, drugs or devices bought for respiratory symptoms specifically, the number of days being absent from work because of respiratory symptoms, as well as any change in employment status caused by respiratory symptoms. This questionnaire is a tool to evaluate direct and indirect costs of WRA during the year preceding the diagnosis of WRA. Along with the questionnaire, the subjects are given a notebook to have them note each month for one year all forms of expenses relative to their occupational asthma (medication, physician visits, hospitalizations, absences from work, cost of making changes to house…), repaid or not by Social Security or their health insurance. This notebook is used as a reminder for the subject, who is phone-interviewed one year later and asked about all their asthma-related expenses and changes in employment status during the year following the diagnosis of WRA. This enables us to evaluate more precisely the direct and indirect costs (for the subject himself or their family, their company or the health system) of WRA during the year following the diagnosis. Another part of the phone interview will consist in the WTP scenario to quantify intangible costs of WRA. The subject will be asked how much they would pay for a drug that would cure their asthma for good, although this drug is more expensive than what the subject pays for their current treatment and is not repaid by the Social Security at all.

### Implementation aspects, logistics and first results

Based on the estimation that about 5% of the general population have an active asthma – with a crisis during the past 12 months [31], and that occupational physicians can see about 100 workers over a 2-week period, 100 screening questionnaires and 5 data collection books as well as 5 peak-flow meters are sent to each participating occupational physician. A protocol for the physicians to follow, an information note for the workers, and pre-paid envelopes to send the questionnaires back are joined too. Four occupational physicians and the workers they monitor and Nancy’s UHODD and their patients with suspected WRA have tested our screening questionnaire and epidemiological data collection book. To help the subject take PEF measurements and fill out the diary, we published an instructional video on Youtube (http://youtu.be/9vLKrygWQWc). Additional epidemiological data collection books and peak-flow meters are sent to the occupational physicians if they estimate more than five are necessary. Screening questionnaires are entered in database by optical scanning device, and technicians enter other questionnaires and PEF measurements in the computer system.

At the 1^st^ of July 2015, after 6 months, 17 physicians included 1139 subjects in step 1, with 144 potential asthmatics who received the PEF meter and the questionnaires, among which 38 have returned the epidemiological data collection book. Of these, 28 were staffed at phase 2: 3 were classified as “probably without asthma”, for 2 we had no suspicion of WRA and 23 were classified as potential WRA. At this stage, none had a final step 3 diagnosis.

## Discussion

The presented project is an attempt to obtain a global picture of occupational asthma in France. Consequently it covers many aspects and consequences of work-related asthma in a holistic approach. This includes clinical aspects for the precise diagnosis, public health aspects in assessing the prevalence of asthma globally and the different subtypes of work-related asthma in the working population with their corresponding phenotypes as well as social aspects by assessing both the direct and indirect cost of WRA. To set up this project, a multidisciplinary research group was set up including epidemiologists, occupational health specialists, chest physicians, and economists. This study has several strengths some of which are due to the dual recruitment. Thus, most of the WRA cases seen in a clinical setting are OA, as these are more readily identified as work-related. Moreover, most of these cases are due to standard asthmogens [32] (flour, persulfate salts, isocyanates…), so that they are easier to detect by occupational physicians or chest physicians. Moreover the severity of asthma is probably more important among clinical cases. The systematic screening of active workers is thus expected to yield more WEA cases but less severe cases. On the other hand this screening might detect an increased asthma prevalence in sectors not exposed to standard asthmogens, as compared with a clinical setting. As step 1 consists of the systematic recruitment of workers, every worker will fill in at least the screening questionnaire, already giving us some data about the potential presence of symptoms of an active asthma, the evolution of the symptoms when away from work and the professional occupation. Hence, even for subjects not agreeing to participate in step 2 or 3, we will be able to have some information that can be used to estimate baseline prevalence of asthma-like symptoms in a working population. As a side-effect this screening will allow us to include asthmatics (both non-work-related and work-related) that may have not been detected and might thus get a better medical follow-up. All this will enable us to estimate more accurately the prevalence of WRA in a working population. A first product of this project is in the standardized procedure developed for the differential diagnosis of WRA that is designed to yield reproducible and comparable results between clinical centers with different diagnostic tools. This study will enable us to test the feasibility and reproducibility of the procedure as well as its sensitivity and specificity with respect to a consensus statement of a group of specialized clinicians. If validated, this procedure could be used later as a harmonized routine procedure in all UHODDs and be proposed as a standard “objective” diagnostic. A final strength is the economical part of our project. As the estimation of the costs involved will be based on a standardized diagnosis with through the epidemiological part, it would allow us a better estimation of the global cost of WRA as it would include an estimation of the cost of the WRA cases which would not have been recognized as such without a systematical screening study such as the one we conduct in this project. Of particular interest, though a side-issue in this global estimation, is the estimation of the contingent costs, through as self-declared “Willingness to pay” approach, which could be considered as a self-perceived control. This project is however not exempt of weaknesses: our study relies on occupational physicians’ and subjects’ willingness to participate so that the recruitment is very work-intensive and our expected number of subjects might be difficult to meet. A second drawback, related to the first, is that despite the large expected population to be included, this population cannot be considered representative of the total French workforce. Indeed, some sectors (agriculture, craftsmen…) are not followed up by occupational physicians, which limits the comparison with the source population of the UHODDS. As a result, the prevalence of asthma in the epidemiological part is estimated only out of a part of French industries. It must also be stressed that for all subjects who do not participate in the clinical phase (Step 3), the final classification of subjects into groups (OA, WEA, NWRA, no asthma) is based on their answers to self-administered questionnaires and peak-flow measurements during 2 weeks only, so that no final diagnosis can be made. However, sensitivity was privileged in the detection of WRA: any suspicion of work-relation leads to classification as potential WRA. With respect to the asthma severity: according to the GINA consensus (Global strategy for asthma management and prevention) [10], it should be assessed once the subject has been on controlled treatment for several months, it is thus not estimable from the present study. In our population, subjects are at different therapeutical steps, which does not enable us to assess severity levels according to GINA’s definition. However, exacerbations frequencies and symptoms, non-specific bronchial hyperreactivity and bronchial inflammation intensity are taken into account. It must be noted, though, that our study cannot be considered as an etiological study, as the precision of the exposure assessment varies greatly between the three steps of our assessment. Finally, in this study we identify an important number of asthmatic and non-asthmatic subjects who agreed to participate. This database could be the basis of ancillary studies, such as exploring etiological aspects by comparing the occupational settings between the different groups of subjects having similar personal factors. A longitudinal follow-up could assess the effectiveness of an early screening of the disease. Moreover, the possibility of getting genetic material would allow to study gene-environment interactions (GEI).

The occupational setting-based recruitment of the epidemiological part of the study will enable us to estimate more accurately the prevalence of WRA in a working population. The diagnosis procedure elaborated for the clinical part of the study could be proposed as a standard objective diagnostic procedure if validated. Due to a combined epidemiological and clinical protocol with a multidisciplinary approach, this project covers a broad series of issues regarding WRA.

## Additional file

Additional file 1: This file presents the items used to determine the status of potential asthmatic subject in a short table. (PDF 22 kb)
