# Supplementary material for: Assessment of work-related asthma prevalence, control and severity: protocol of a field study
Source: BMC Public Health. 2016 Nov 16;16:1164. doi: 10.1186/s12889-016-3824-0 (PMC5112681; doi:10.1186/s12889-016-3824-0)
Supplement: Additional file 1: — This file presents the items used to determine the status of potential asthmatic subject in a short table. (PDF 22 kb) [file 12889_2016_3824_MOESM1_ESM.pdf]

Additional file 1 – Items used to determine the status of potential asthmatic subject

1. Are you currently taking asthma medication?	Yes ☐	No ☐
2. At any time in the last 12 months, have you:		
a) had an asthma attack?	Yes ☐	No ☐
b) had wheezing in your chest?	Yes ☐	No ☐
c) been woken up with a feeling of tightness in your chest first thing in the morning?	Yes ☐	No ☐
d) had an attack of shortness of breath that came on during the day when you were not doing anything strenuous?	Yes ☐	No ☐
e) had wheezing in your chest after doing something strenuous?	Yes ☐	No ☐
f) been woken up at night by an attack of shortness of breath?	Yes ☐	No ☐
g) been woken up at night by a coughing fit?	Yes ☐	No ☐
3. During your work shift or within the following few hours, do you happen to:		
a) start to cough?	Yes ☐	No ☐
b) start having wheezing in your chest when breathing?	Yes ☐	No ☐
c) have shortness of breath, a feeling of oppression or tightness in your chest?	Yes ☐	No ☐
